# FeedER: a feedback-regulated enzyme-based slow-release system for fed-batch cultivation in microtiter plates

**DOI:** 10.1007/s00449-019-02180-z

**Published:** 2019-08-09

**Authors:** Roman Jansen, Niklas Tenhaef, Matthias Moch, Wolfgang Wiechert, Stephan Noack, Marco Oldiges

**Affiliations:** 1 Forschungszentrum Jülich, Institute of Bio- and Geosciences, Biotechnology (IBG-1), Jülich, Germany; 2 RWTH Aachen University, Computational Systems Biotechnology (AVT.CSB), Aachen, Germany; 3 Institute of Biotechnology, RWTH Aachen University, Aachen, Germany; 4 Bioeconomy Science Center (BioSC), Forschungszentrum Jülich GmbH, Jülich, Germany

**Keywords:** FeedER, Exponential fed-batch, Miniaturized cultivation, *C. glutamicum*, High-throughput bioprocess development, Online monitoring

## Abstract

With the advent of modern genetic engineering methods, microcultivation systems have become increasingly important tools for accelerated strain phenotyping and bioprocess engineering. While these systems offer sophisticated capabilities to screen batch processes, they lack the ability to realize fed-batch processes, which are used more frequently in industrial bioprocessing. In this study, a novel approach to realize a feedback-regulated enzyme-based slow-release system (FeedER), allowing exponential fed-batch for microscale cultivations, was realized by extending our existing Mini Pilot Plant technology with a customized process control system. By continuously comparing the experimental growth rates with predefined set points, the automated dosage of Amyloglucosidase enzyme for the cleavage of dextrin polymers into d-glucose monomers is triggered. As a prerequisite for stable fed-batch operation, a constant pH is maintained by automated addition of ammonium hydroxide. We show the successful application of FeedER to study fed-batch growth of different industrial model organisms including *Corynebacterium* *glutamicum*, *Pichia* *pastoris*, and *Escherichia* *coli*. Moreover, the comparative analysis of a *C.* *glutamicum* GFP producer strain, cultivated under microscale batch and fed-batch conditions, revealed two times higher product yields under slow growing fed-batch operation. In summary, FeedER enables to run 48 parallel fed-batch experiments in an automated and miniaturized manner, and thereby accelerates industrial bioprocess development at the screening stage.

**Electronic supplementary material:**

The online version of this article (10.1007/s00449-019-02180-z) contains supplementary material, which is available to authorized users.

## Introduction

The last decade is marked by tremendous advances in the development of highly engineered strains for bioprocess application. Through the emergence of specific gene editing tools in combination with high-throughput screening tools such as biosensors, large strain libraries can be generated within a short period of time [1–3]. To determine the best candidates for process development, these libraries need to be characterized in detail, and thus the number of necessary experiments can easily sum up to a thousand per library [4]. Consequently, there is a great demand for systems allowing an increased experimental throughput without sacrificing data accuracy and reproducibility.

Various stand-alone microbioreactor (MBR) systems have evolved in the last years, allowing strain phenotyping with an adequate throughput without loss of control and monitoring capabilities [5]. To further increase phenotyping capacity and decrease manual handling steps, these MBR systems are often embedded into robotic platforms. The parallelized cultivation in combination with a liquid handling system allows the full pipeline from automated inoculation, dosing, and sampling procedures followed by analytics [6].

The current bottleneck of these platforms is the mode of cultivation, i.e., they are mostly restricted to batch operation. However, fed-batch operation is preferred in industrial production to prevent, e.g., oxygen limitation, substrate inhibition, and overflow metabolism [7], and to achieve higher product titers and yields. Screening in batch mode might also lead to wrong strain selections due to artificial environmental conditions [8]. Consequently, multiple approaches to also enable fed-batch cultivation on the available MBR platforms have been developed.

For example, in combination with a liquid handling system, a dissolved oxygen (DO)-triggered fed-batch can be realized. Every time the DO signal spikes, indicating the complete consumption of all carbon sources, new substrate can be added via the robot [9]. However, this creates an oscillating effect on the cell metabolism, and thus the obtained results are not fully comparable to bioreactor fed-batch cultivations.

Alternatively, diffusion-based fed-batch operation can be achieved through the addition of silicone elastomer discs containing the primary substrate [8, 10]. As an extension, specialized MTPs with hydrogel channels connected to a substrate reservoir can be applied for a diffusion-based release [11]. On the other hand, enzymatic release of d-glucose from a carbohydrate polymer allows MBR fed-batch cultivation, which is limited to a low release rate without pH control. However, through variation of enzyme concentration, the release rate can be changed [12–14]. Mixed substrate feeding might be beneficial when working with organisms such as *Pichia pastoris* to avoid substrate inhibition [15]. A major disadvantage of all these technologies is the lack of control for the substrate release rate during a fed-batch process. An automated process with addition of enzyme, carbohydrate polymer as well as free d-glucose has been established for online experimental redesign, and thus parameter estimation [16]. Although this process can be used for parameter estimation of a known model, it might be unsuitable for accelerated phenotyping experiments, e.g., due to a lack of valid bioprocess models.

Microfluidic MBR systems utilizing microchannels and microvalves for substrate feeding are another promising alternative [17–19]. However, these systems are still technically challenging. A parallel investigation toward the necessity of pH control in MTPs was conducted, however, without full automation and with no application of any fed-batch strategies [20].

In this study, we present an advanced approach for the utilization of slow-release systems to achieve exponential growth rates during fed-batch microscale cultivation. In particular, the substrate release rate and, therefore, the growth rate is feedback controlled based on online biomass measurements and automated pH control. We show the general applicability of our microscale fed-batch approach using the model organisms, *Corynebacterium* *glutamicum*, *Pichia* *pastoris*, and *Escherichia* *coli*. Additionally, we studied the impact of different growth rates on the formation of the model product GFP with *C.* *glutamicum*.

## Materials and methods

### Chemicals

All chemicals (analytical grade) were purchased from Carl Roth (Karlsruhe, Germany) or Sigma-Aldrich (Steinheim, Germany).

### Bacterial strains and medium

*Corynebacterium glutamicum* strain ATCC 13032 was used. The strain expresses and secretes a GFP-fusion protein pCGPhoD^Cg^-GFP under the control of an IPTG-inducible promoter of the pEKEx2 plasmid with kanamycin resistance [21]. The PhoD signal peptide allows for fully folded GFP secretion utilizing the Tat-pathway. Kanamycin was added at a concentration of 25 mg L^−1^. The strain was stored at − 80 °C via cryo-conservation. A defined CGXII medium [22] was slightly adapted and used for cultivation. The medium contained per liter of deionized water: 1 g K_2_HPO_4_, 1 g KH_2_PO_4_, 13.25 mg CaCl_2_ * 2 H_2_0, 0.25 g MgSO_4_ * 7 H_2_O, 10 mg FeSO_4_ * 7 H_2_0, 10 mg MnSO_4_ * 4 H_2_O, 0.313 mg CuSO_4_ * 5 H_2_0, 0.02 mg NiCl_2_ * 6 H_2_O, 1 mg ZnSO_4_ * 7 H_2_O, 0.2 mg biotin, 30 mg protocatechuic acid, and 5 mL 2.5% (m v^−1^) NH_3_. MOPS, urea, d-glucose, and dextrin from potato starch (Sigma-Aldrich, Steinheim, Germany) were added in varying concentrations for each experiment. The components were prepared as stocks, sterilized separately, and added immediately before the cultivation. The vitamin and protocatechuic acid stocks were aliquoted and stored at − 20 °C to ensure identical conditions for each experiment. For slow-release cultivations, a 300 U L^−1^ stock of Amyloglucosidase from *A. niger* (Sigma-Aldrich, Steinheim, Germany) was stored at 4 °C on the robotic workspace and added as needed by the liquid handling system to the cultivation.

*E. coli* K12 MG1655 wild-type strain was cultivated in slightly modified Wilms-MOPS medium [23]. The medium contained per liter of deionized water: 5 g (NH_4_)_2_SO_4_, 0.5 g NH_4_Cl, 3 g K_2_HPO_4_, 2 g Na_2_SO_4_, 0.5 g MgSO_4_ * 7 H_2_O, 10.46 g MOPS, 0.01 g thiamine hydrochloride, 0.54 mg ZnSO_4_ * 7 H_2_O, 0.48 mg CuSO_4_ * 5 H_2_O, 0.3 mg MnSO_4_ * H_2_O, 0.54 mg CoCl_2_ * 6 H_2_O, 41.76 mg FeCl_3_ * 6 H_2_O, 1.98 mg CaCl_2_ * 2 H_2_O, and 33.39 mg Na_2_EDTA. 35 g dextrin and 5 d-glucose were added as the sole carbon sources. The components were prepared as stocks, sterilized separately, and added immediately before the cultivation. For the cultivation of *P. pastoris* X33, a different chemically defined medium was slightly adapted and utilized [24]. The medium contained per liter of deionized water: 7.5 g (NH_4_)_2_SO_4_, 1 g MgSO_4_ * 7 H_2_O, 8.5 g KH_2_PO_4_, 0.05 mg biotin, 1 mg Ca d-panthothenate, 1 mg nicotinic acid, 25 mg myo-inositol, 1 mg thiamin hydrochloride, 1 mg pyridoxol hydrochloride, 0.2 mg p-amino benzoic acid, 30 mg Na_2_EDTA, 9 mg ZnSO_4_ * 7 H_2_O, 1.55 mg MnCl_2_ * 6 H_2_O, 0.6 mg CuSO_4_ * 5 H_2_O, 0.8 mg Na_2_MoO_4_ * H_2_O, 9 mg CaCl_2_ * 2 H_2_O, 6 mg FeCl_2_ * 7 H_2_O, 2 mg H_3_BO_3_, 0.2 mg KI, and 10.46 g MOPS. 35 g Dextrin and 5 d-glucose were added as the sole carbon sources. The components were prepared as stocks, sterilized separately and added immediately before the cultivation.

### Microbioreactor cultivation

Cultivations were performed utilizing BioLector devices (m2p-labs GmbH, Baesweiler, Germany). FlowerPlates as characterized by Funke et al. [25], commercialized by m2p-labs (MTP-48-BO), and sealed with a sealing foil for automation (F-GPRS48-10) (all by m2p-labs GmbH, Baesweiler, Germany) were used. The medium was inoculated directly from a frozen cryo-culture to an OD_600_ of 0.1. The cultivation was performed at 1300 rpm with an orbital shaking diameter of 3 mm, the humidity set above 85%, and an initial filling volume of 800 µL. The biomass, dissolved oxygen, and pH were measured non-invasively throughout the cultivation. The BioLector is integrated into a liquid handling system (EVO 200) provided by Tecan (Männedorf, Switzerland) with eight steel needle tips, allowing for automated dosing and sampling into each well of the microtiter plate.

### Process control system

To realize the feedback-regulated slow-release fed-batch system, a tailor-made, python-based process control system was employed. The program reads and processes BioLector data during the experiment. The current growth rate for each cultivation is determined by performing a spline approximation of the blanked backscatter data points obtained from the last 30 min. The first derivative of the resulting spline is evaluated at the input time points. The resulting vector is then divided by the blanked backscatter data points. The average of this vector serves as an estimation for the current growth rate.

Based on that rate, the decision to add enzyme is made. At first, end of the initial batch is detected (growth rate < 0.05 h^−1^). This triggers the addition of the initial dose of enzyme. After starting the fed-batch, the following procedure is done: if the growth rate falls under a set threshold, the enzyme is added to the cultivation using the liquid handling system. The amount of added enzyme is determined by multiplication of the current backscatter value with a factor to compensate necessary increase of release rate at higher biomass concentration. For pH control, the measured pH is monitored. If the pH drops below 7.1, 10 µL of 2.5% (w v^−1^) NH_3_ is added using the liquid handling system. In addition to controlling the process, the program also provides the user with plots of the relevant data during the process.

### Product analysis

The secreted GFP was analyzed via optical measurements performed by the BioLector with LED/filter module for fluorescence measurement.

## Results and discussion

### Automated pH control for microtiter plate cultivation

**Fig. 1:**
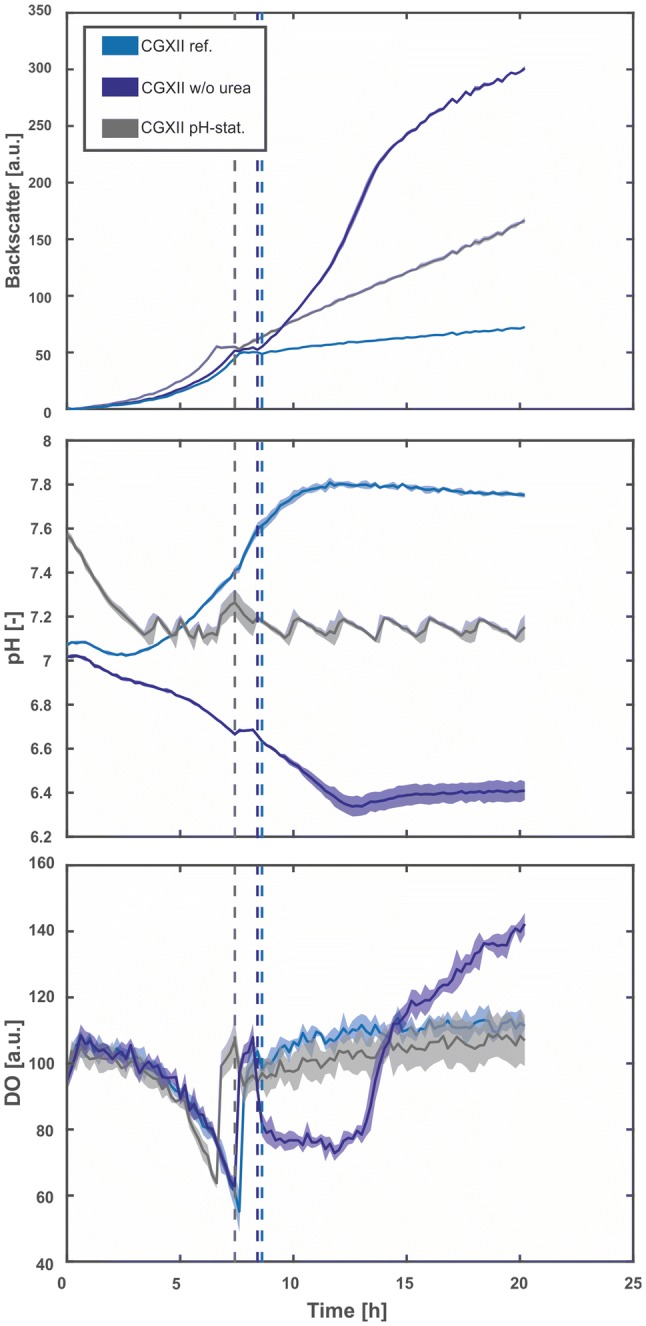
Implementation of a pH control for microscale fed-batch cultivation. Cultivations were performed in a 48-well FlowerPlate under standard conditions with 5 g L^−1^d-glucose and 35 g L^−1^ dextrin as the carbon sources. Solid lines represent the arithmetic means of six biological replicates and shaded areas represent the corresponding standard deviations. The light blue lines show measurements of the cultivation in CGXII medium with 5 g L^−1^ urea and 200 mM MOPS, but without any pH control (condition A). The dark blue lines show the measurements for condition B, where CGXII medium without urea was used. The grey lines show condition C: cultivation without urea, only 50 mM MOPS and automated pH control. A threshold for the pH was set at 7.1. When the online signal dropped below the set point, 10 µL of 2.5% (m v^−1^) NH_3_ was added to the cultivation by the liquid handling system. The dotted lines indicate the time points when the enzyme was pulsed into the media (color figure online)

The slow-release system used in this study is based on the conversion of a dextrin polymer to d-glucose by the enzyme Amyloglucosidase from *Aspergillus niger*. To establish feedback-controlled enzyme release-based fed-batch cultivations, it is essential that the d-glucose release rate is only dependent on the enzyme activity modulated by the amount of enzyme in the cultivation media. However, the activity of Amyloglucosidase is strongly dependent on the pH. For example, it was shown by Toeroek et al. that the activity increased by 40% when the pH was changed from 7.3 to 6.8 [26]. Therefore, for a controllable enzymatic release rate of d-glucose, a constant pH is an essential prerequisite. This constant pH may be provided by the standard defined medium for *C. glutamicum*, CGXII [22], which contains 200 mM MOPS as buffer system. A different strategy is based on an automated, one-sided pH control using the capabilities of a liquid handling system. We compared both strategies by performing eight replicate cultivations of *C.* *glutamicum* wild-type under three different conditions (Fig. 1).

As a reference (condition A), CGXII medium with 83.25 mM urea and 200 mM MOPS was used and no external pH control was applied. In condition B, CGXII medium without urea was tested, again without external pH control. For automated pH control (condition C), we modified the CGXII medium to implement a control strategy as follows: first, to enable a one-sided control strategy, no urea was added. Second, to achieve a more sensitive response, the buffer capacity was reduced to 50 mM MOPS, also lowering osmotic pressure. Third, when the online-measured pH drops below a set point of 7.1, 10 µL of 2.5% (m v^−1^) NH_3_ was added by the liquid handling system.

For all conditions, 5 g L^−1^d-glucose was added to the medium for an initial batch phase. For the fed-batch phase, 35 g L^−1^ dextrin was added, which was then converted to d-glucose monomers through addition of Amyloglucosidase. The end of the initial batch phase was detected automatically via at-line analysis of the growth rate. Subsequently, an initial single pulse of Amyloglucosidase was automatically added into each well to start the fed-batch phase (represented as dotted lines in Fig. 1).

For cultivations in condition A, the pH strongly increased from 7.1 to 7.8 due to the assimilation and degradation of urea in the initial batch phase. Even in the higher buffered condition with 200 mM MOPS, this effect could not be prevented. At this pH, the activity of the d-glucose-releasing enzyme is very low. Consequently, only a slight increase in backscatter was observed in the fed-batch phase.

On the contrary, for condition B, where no urea was added to the medium, the pH decreased throughout the initial batch phase from 7.0 to 6.7 due to ammonium uptake. After addition of the enzyme, the backscatter signal increased rapidly as expected, indicating fast biomass growth caused by release of d-glucose. However, the pH continued to decrease during fed-batch phase, which in turn further increased the activity of the enzyme. As a result, the growth curve did not follow the intended linear trend, but accelerated in a rather unpredictable manner. The pH drop seems to be a consequence of slight acidification which goes along with the utilization of d-glucose and is again not fully compensated by the buffered condition. This renders our approach for controlled exponential fed-batch cultivation impossible.

In condition C, the automated pH-control for the modified CGXII medium worked as expected. Throughout the cultivation, the control strategy kept the pH between 7.1 and 7.3. After the initial batch phase and addition of Amyloglucosidase, a linear increase of backscatter was observed, indicating that the d-glucose release rate was indeed kept constant by maintaining a constant pH. The small oscillation of pH does not seem to hamper achieving a linear growth profile. With this setup at hand, development of an exponential fed-batch system was started.

### Feedback-regulated enzyme-based slow-release fed-batch cultivation (FeedER)

FeedER is built on the ability to calculate the current growth rate during cultivation. This is done by utilizing the non-invasive biomass measurement via backscatter and appropriate processing of the signal via spline approximation as described previously [27].

A drop in the current growth rate below a predefined threshold triggers the addition of Amyloglucosidase. By adding this enzyme, the overall d-glucose release rate is increased. The amount of enzyme to be added is dependent on the current biomass concentration and, therefore, has to be accordingly increased along the cultivation. The initial dosing volume was 5 µL and reached up to 25 µL per dose. To account for the changing volume due to the addition of enzyme and base, the backscatter signal is volume corrected.

**Fig. 2:**
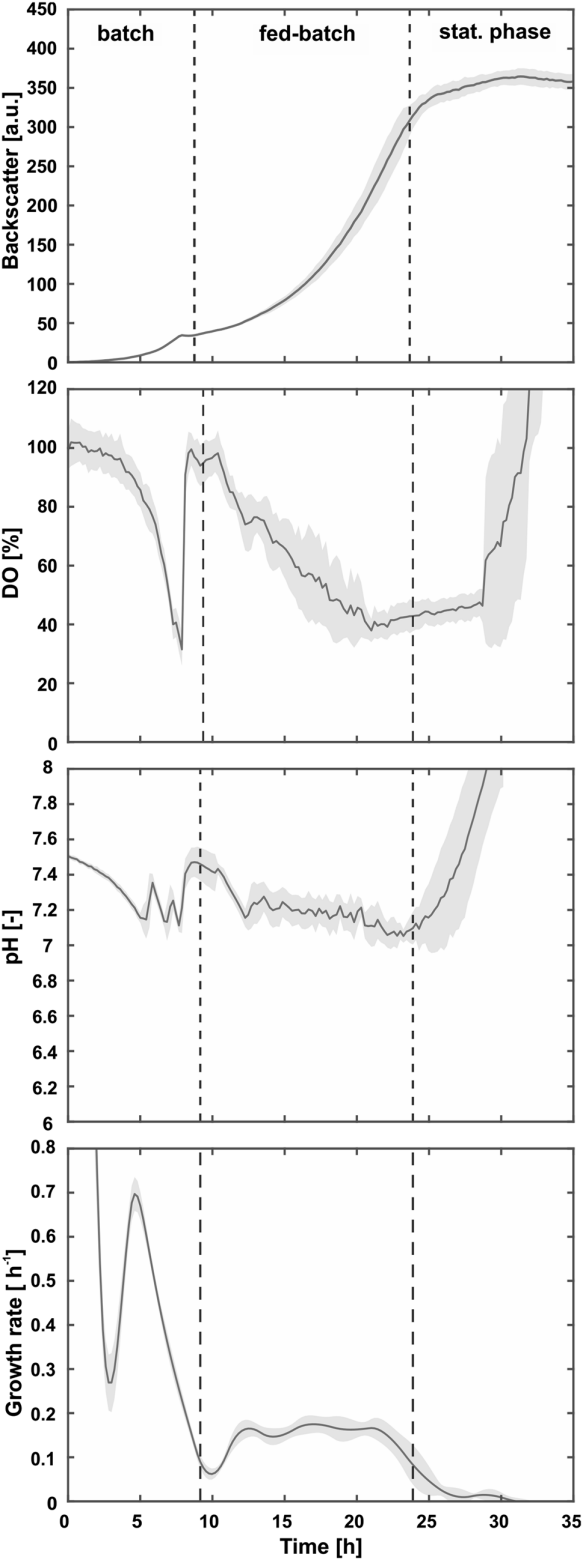
Exponential fed-batch culture of *C.* *glutamicum* ATCC 13032 with enzyme addition at a growth rate threshold of *µ*_set_ = 0.1 h^−1^. Cultivations were performed in a 48-well FlowerPlate at 30 °C, 1300 rpm, > 85% humidity, and the initial filling volume was 800 µL. Modified CGXII medium with 5 gL^−1^d-glucose, a dextrin equivalent of 75 g L^−1^d-glucose and 50 mM MOPS were used. The pH was kept constant at 7.1 through addition of 2.5% (m v^−1^) NH_3_. The growth rate was kept above the set point through specific addition of Amyloglucosidase. Dark lines represent the arithmetic means of 12 biological replicates and shaded areas represent the corresponding standard deviations

We tested this strategy by cultivation of *C.* *glutamicum* ATCC 13032 in modified CGXII medium as described in Sect. 3.1. As a first set point for the growth rate (*µ*_set_) triggering enzyme addition, we applied *µ*_set_ = 0.1 h^−1^ (Fig. 2). After an initial batch phase on 5 g L^−1^d-glucose, the fed-batch cultivation was started through addition of the first dose of Amyloglucosidase. During the fed-batch, further enzyme was added, if the growth rate dropped below the threshold as described above. The pH was kept constant between 7.1 and 7.3 through the addition of 2.5% (m v^−1^) NH_3_. The fed-batch cultivation was stopped when the particular well reached a maximal filling volume of 1200 µL. An exemplary dosing history can be found in Fig. S3.

The novel strategy allows for a highly reproducible fed-batch cultivation, as demonstrated by the small standard deviations for all signals (cmp. shaded areas in Fig. 2). The average experimental growth rate for 12 biological replicates during the fed-batch phase was *µ*_exp_ = 0.14 ± 0.01 h^−1^. The resulting deviation from the set point can be explained by the implemented dosing procedure. Each time the measured growth rate drops below the threshold, new enzyme was added to increase the growth rate accordingly. A posteriori calculation of the growth rate during the fed-batch phase, therefore, yields a value higher than the threshold defined. The increase of pH after 25 h might be due to metabolization of small amounts of acidic by-products after depletion of the main carbon source d-glucose. This is consistent with a steady DO signal, which took until all carbon was depleted and the DO signal rises again.

**Fig. 3:**
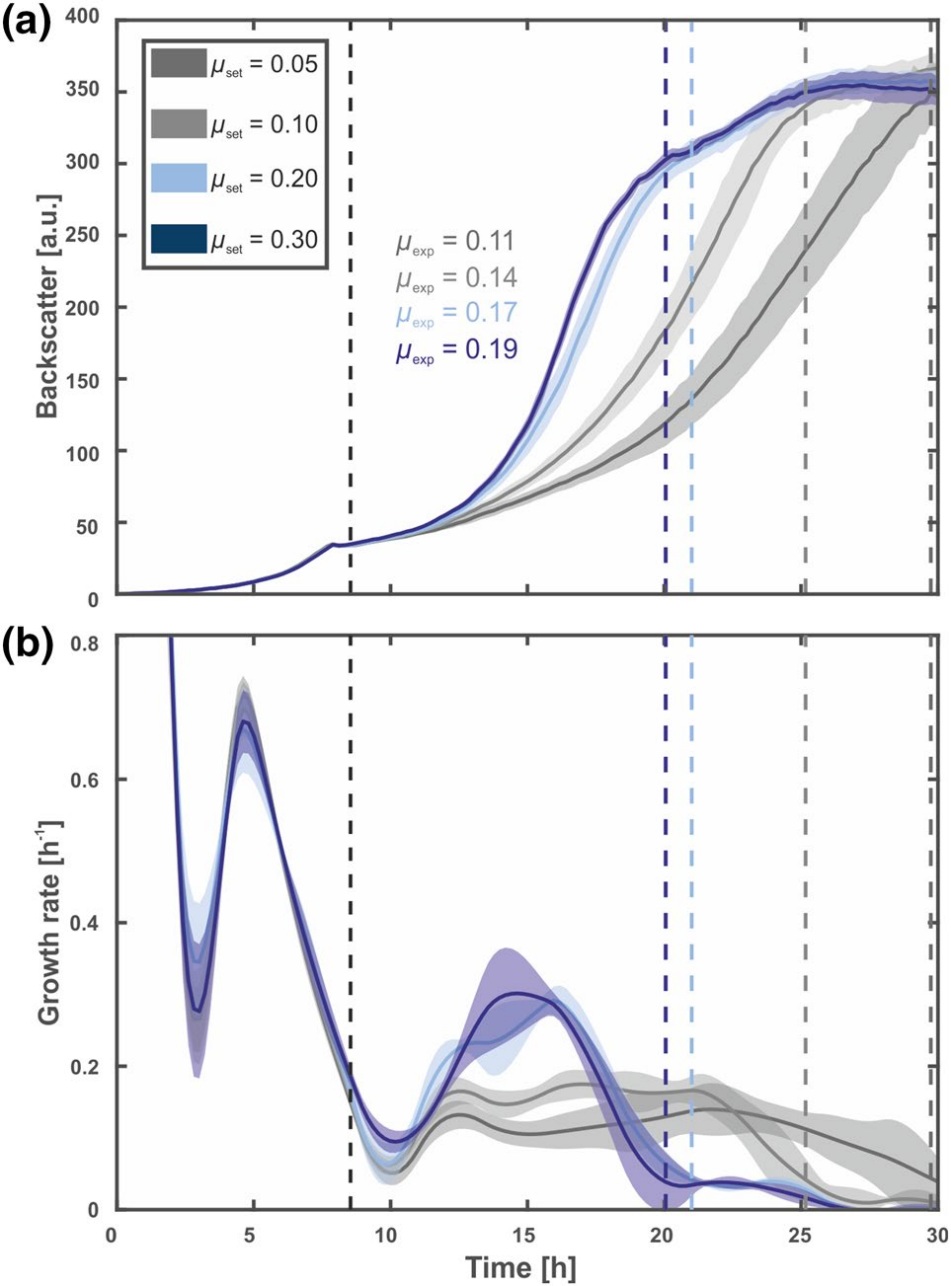
Exponential fed-batch cultures of *C. glutamicum* ATCC 13032 with enzyme addition at four different growth rate thresholds. Cultivations were performed in a 48-well FlowerPlate at 30 °C, 1300 rpm, > 85% humidity, and the initial filling volume was 800 µL. Modified CGXII medium with 5 g L^−1^d-glucose, a dextrin equivalent of 75 g L^−1^d-glucose and 50 mM MOPS were used. The pH was kept constant at 7.1 through addition of 2.5% (m v^−1^) NH_3_. Four different growth rate set points *µ*_set_ = 0.05, 0.1, 0.2, and 0.3 h^−1^ were tested. The growth rates were kept above the set points through specific addition of Amyloglucosidase. Dark lines represent the arithmetic means of 12 biological replicates and shaded areas represent the corresponding standard deviations. The cultivations were stopped when a maximal filling volume of 1200 µL was reached. **a** shows the volume corrected backscatter signal and **b** shows the growth rate calculated via spline approximation after the experiment. The dotted lines represent the start of the fed-batch and the end point, determined when the growth rate dropped below 0.05 h^−1^

We continued to test our approach with three other growth rate set points, i.e., *µ*_set_ = 0.05, 0.20, and 0.30 h^−1^, respectively. The results are shown in Fig. 3. The growth rate during the fed-batch phase was calculated from the first enzyme dosing up the point where the calculated growth rate dropped below a threshold of *µ*_exp_ = 0.05 h^−1^, indicating the end of the fed-batch phase. The lowest set point of *µ*_set_ = 0.05 h^−1^ consequently resulted in the slowest biomass increase during fed-batch cultivation and the longest fed-batch phase of 20 h. Moreover, the increase in *µ*_set_ = 0.1 and 0.2 h^−1^ also resulted in faster biomass accumulation with *µ*_exp_ = 0.14 h^−1^ and 0.17 h^−1^, respectively. Strikingly, set points of *µ*_set_ = 0.2 and 0.3 were yielding very similar experimental growth rates (Fig. 3b).

This experiment demonstrated that a certain range of growth rates can be realized with the developed system. Especially in the beginning of the fed-batch phase and for the two set points *µ*_set_ = 0.05 and 0.1 h^−1^, differences in growth can be seen. However, after 10 h of the fed-batch phase, growth curves start to slightly deviate from exponential trends indicated by the plateau (Fig. 3a). The reason for this effect probably lies in the applied dosing regime: only the actual biomass was considered when calculating the amount of enzyme to be added for cleaving the dextrin polymer. Therefore, by additionally taking the growth rate set point into account would possibly solve this issue, since a higher amount of enzyme could be added when aiming for higher exponential growth rates during fed-batch operation.

Another challenge is presented by the set point of *µ*_set_ = 0.3 h^−1^: although the system added more enzyme under these conditions, the growth rate could not be increased accordingly. A possible explanation could be that all open sites of the glucose polymer were already occupied by enzyme and consequently, the d-glucose release rate reached its maximum. A possible solution might be the addition of further highly concentrated dextrin, allowing for more open enzyme sites.

**Fig. 4:**
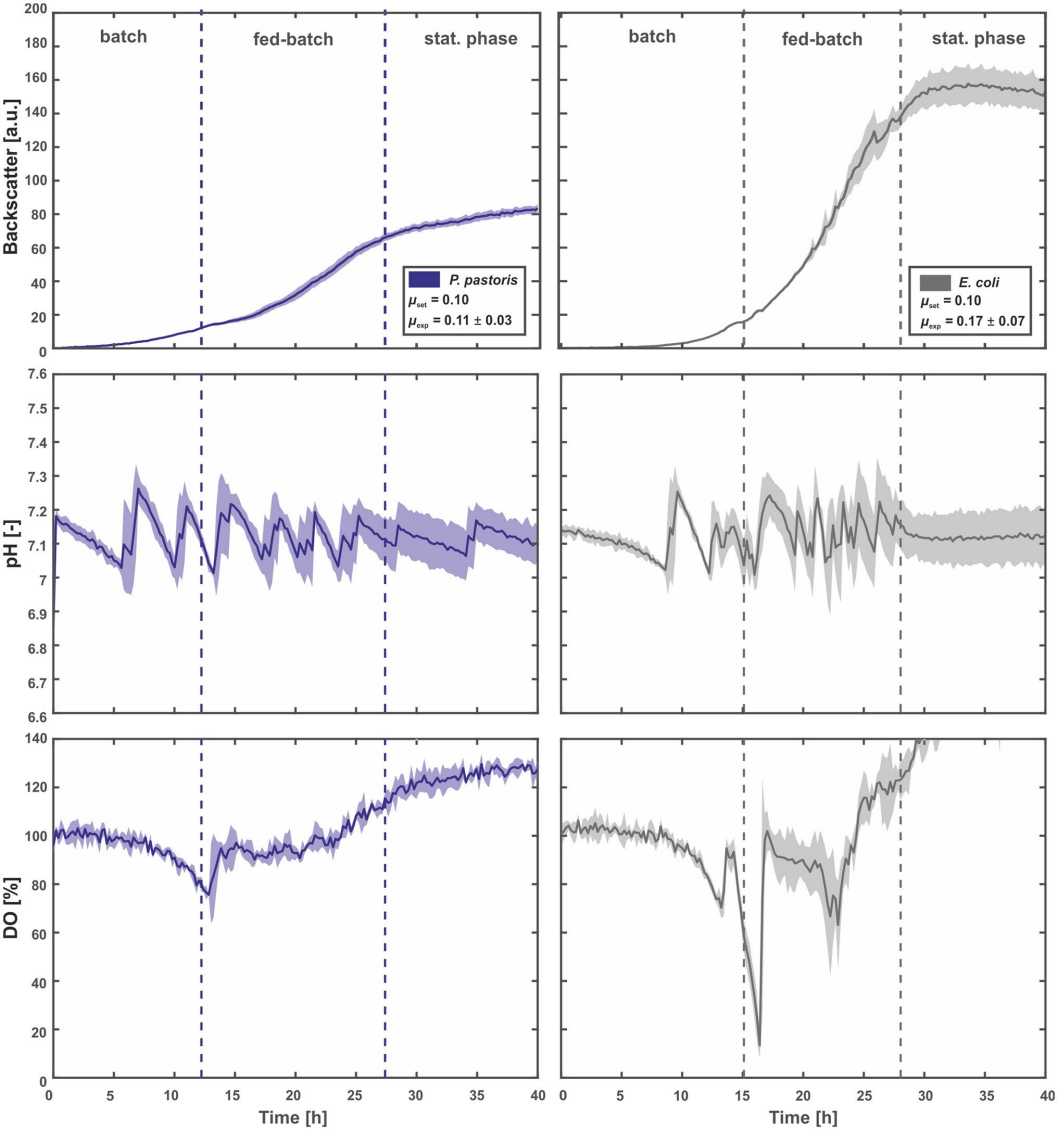
Fed-batch cultivation of *E. coli* and *P*. *pastoris* wild-type strains utilizing the FeedER technology. Cultivations were performed in a 48-well FlowerPlate at 30 °C, 1300 rpm, > 85% humidity, and the initial filling volume was 800 µL. Modified defined media with 5 gL^−1^d-glucose, a dextrin equivalent of 35 g L^−1^d-glucose, and 50 mM MOPS were used. The pH was kept constant at 7.1 through addition of 2.5% (m v^−1^) NH_3_. The growth rate was kept above the set point through specific addition of Amyloglucosidase. Dark lines represent the arithmetic means of 12 biological replicates and shaded areas represent the corresponding standard deviations (color figure online)

To demonstrate the broad applicability of our approach, fed-batch cultivations with *E.* *coli* and *P.* *pastoris* were conducted with *µ*_set_ = 0.1 h^−1^ as the applied threshold. The organisms were cultivated in chemically defined media with 5 g L^−1^d-glucose for an initial batch phase and 35 g L^−1^ dextrin for the subsequent fed-batch phase (Fig. 4 and S4).

Since both microorganisms represent different morphologies, the absolute values of backscatter signals varied greatly. However, both growth curves showed the expected exponential fed-batch profile. While the initial batch phase with consumption of free d-glucose took 12 h for *P. pastoris*, it was 3 h longer for *E.* c*oli*. In both cases, the end of the batch phase was marked by a sudden increase in the DO signal. Interestingly, for *E.* *coli*, a second spike after 17 h occurred, which was most likely due to the consumption of overflow metabolites such as acetate.

The duration of the subsequent fed-batch phases was also different for both organisms, i.e., 15 h for *P.* *pastoris* and 12 h for *E.* c*oli*, respectively. The resulting growth rate of *µ*_exp_ = 0.11 ± 0.03 h^−1^ for *P.* *pastoris* matched almost perfectly with the set point growth rate. For *E.* *coli*; however, the experimentally observed growth rate was significantly higher (*µ*_exp_ = 0.17 ± 0.07 h^−1^). This finding can be explained by the overflow metabolism in the batch phase, which led to a false detection of the end of this phase, i.e., before all metabolites were consumed. As a direct consequence, the first Amyloglucosidase pulse was given too early, leading to a release of d-glucose amount above the level that is required to limit the growth rate at the defined set point.

Nevertheless, both experiments have demonstrated the general applicability of our technology to industrial relevant microorganisms.

### Application of FeedER for studying protein production

In a comparative application study, the strain *C.* *glutamicum* pCGPhoD^Cg^-GFP that is capable of expressing and secreting the model protein GFP was cultivated in both batch and fed-batch conditions. GFP secretion was enabled by Tat translocation using PhoD as Tat-specific signal peptide and its extracellular accumulation was monitored online via fluorescence measurement within the BioLector. The same total amount of d-glucose (80 g L^−1^) was applied for both cultivations and the pH was controlled in an automated manner as described above. For the fed-batch cultures, four different growth rate thresholds for the feedback-regulated process control were set, i.e., *µ*_set_ = 0.05, 0.1, 0.2, and 0.3 h^−1^, respectively.

**Fig. 5:**
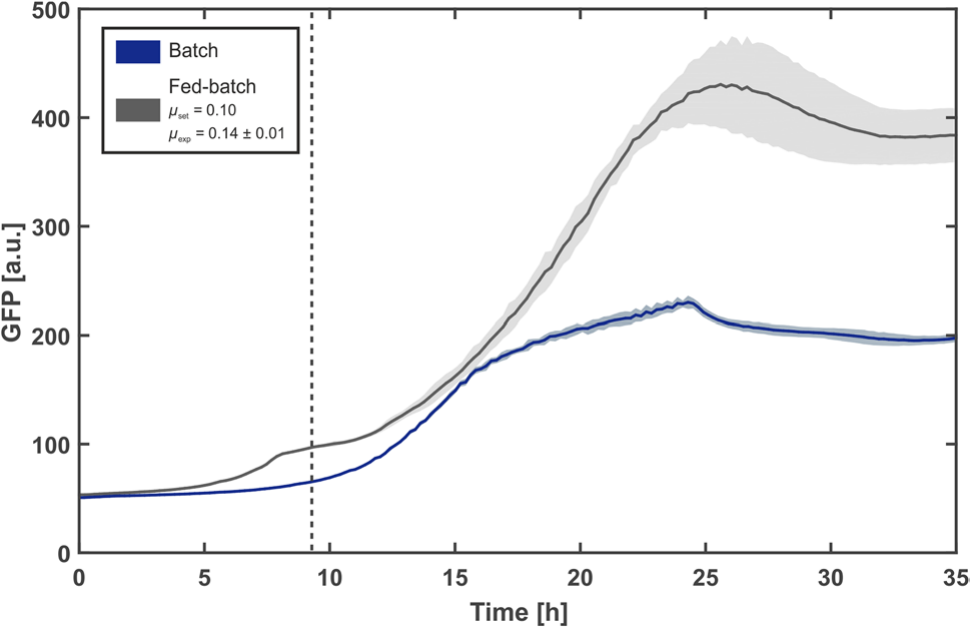
GFP production with *C. glutamicum* pCGPhoD^Cg^-GFP in microtiter plate cultivations. Cultivations were performed in a 48-well FlowerPlate at 30 °C, 1300 rpm, > 85% humidity and the initial filling volume was 800 µL. The batch was performed in standard CGXII with 80 g L^−1^d-glucose. Modified CGXII medium with 5 g L^−1^d-glucose, a dextrin equivalent of 75 g L^−1^d-glucose and 50 mM MOPS was used for the fed-batch cultivation. Here the pH was kept constant at 7.1 through addition of 2.5% (m v^−1^) NH_3_. The growth rate was kept above the set point *µ*_set_ = 0.1 through specific addition of Amyloglucosidase. Dark lines represent the arithmetic means of 12 biological replicates and shaded areas represent the corresponding standard deviations. The secreted GFP production for both batch and fed-batch cultivations were analyzed via fluorescence measurement performed by the BioLector

Figure 5 shows the GFP production in the batch cultivation and an exemplary fed-batch cultivation (*µ*_set_ = 0.1 h^−1^). Data from other cultivations are shown in Fig. S2. Within the batch cultivation, a maximum in GFP titer (216 ± 6 a. u.) was reached after 25 h. In comparison, within the fed-batch cultivation, the amount of secreted GFP was almost doubled (417 ± 76 a. u) at equal cultivation time.

**Fig. 6:**
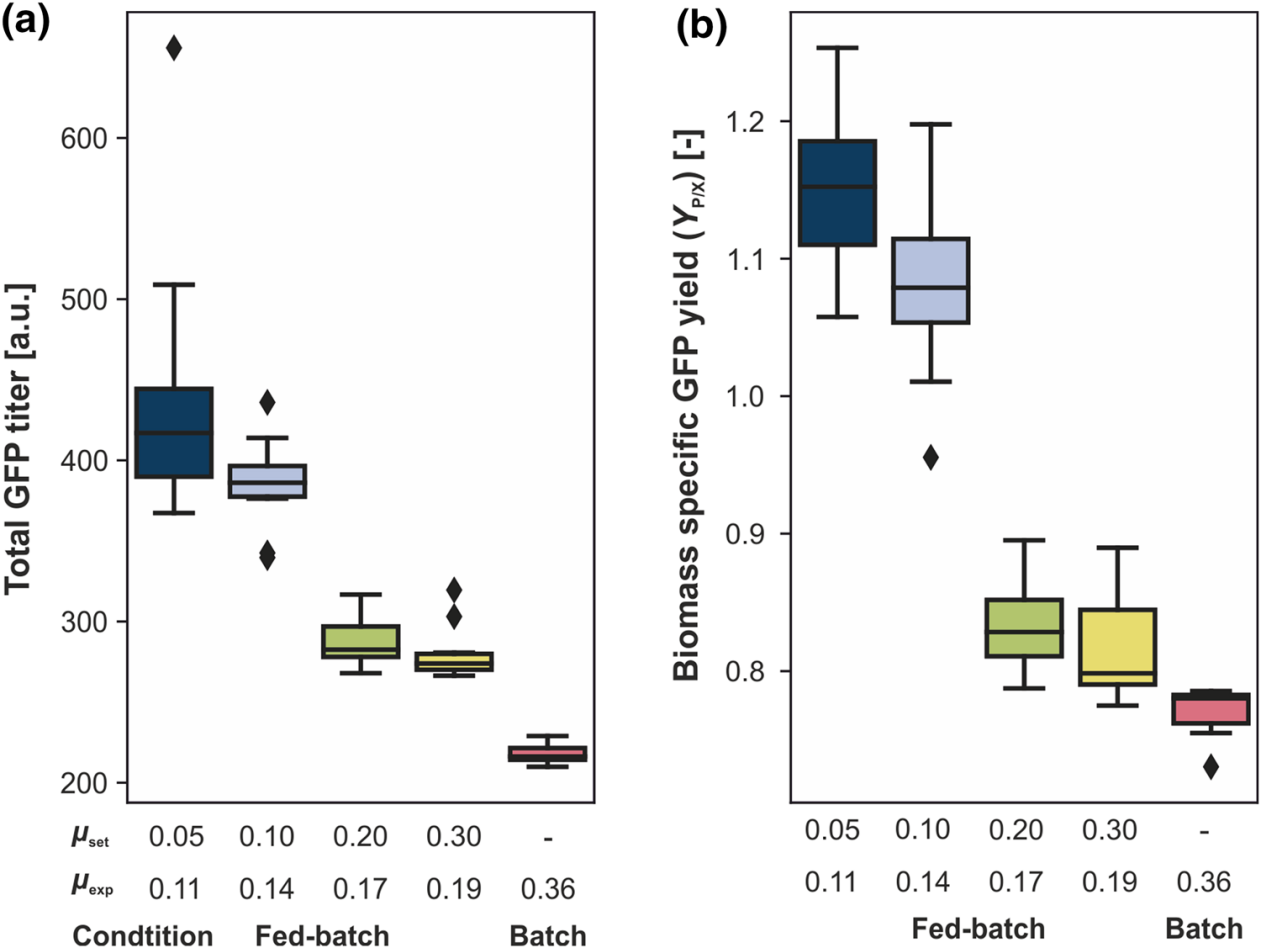
Total GFP titer (**a**) and biomass-specific (**b**) product yield of fed-batch and batch cultivations. Cultivations were performed in a 48-well FlowerPlate at 30 °C, 1300 rpm, > 85% humidity, and the initial filling volume was 800 µL. In all experiments, a total of 80 g L^−1^d-glucose was provided. Biomass specific GFP yield was calculated utilizing the online backscatter and GFP fluorescence signals at the end of the cultivation. Whiskers extend to quartile 1 and quartile 3, respectively. Diamonds mark outliers in the dataset. A point is considered an outlier if its value is higher than Q3 * 1.5 * IQR (interquartile range) for points higher than the median or lower than Q1 * 1.5 * IQR for points lower than the median value

Total GFP titer and substrate-specific product yields were calculated for all cultivations (Fig. 6). Starting from the highest titer and yield (417 and *Y*_P/X_ = 1.15, respectively) at the lowest growth rate (*µ*_exp_ = 0.11 h^−1^), both quantities continuously decreased with increasing growth rates leading to a final reduction of 34% and 31% at the highest rate (*µ*_exp_ = 0.19 h^−1^). This can be explained by the fact that with increasing substrate availability, GFP production is not increased at the same rate as compared to biomass growth. Consequently, the specific product yield dropped. Since the average growth rate of the batch cultivation was the highest, GFP titer and yield were also the lowest. Overall, a lower growth rate during fed-batch cultivation proved to be highly beneficial for GFP production. This correlation has already been reported in the literature for the intracellular production of proteins, such as GFP or nucleases, with *E.* *coli* [28, 29]. More recently, the same observation was made for the secretion of cutinase with *C.* *glutamicum* in a bioreactor fed-batch process [30].

## Conclusion

In this study, we demonstrated the development of FeedER, which is a feedback-controlled slow-release fed-batch system. FeedER is based on automated pH control and addition of Amyloglucosidase, which in turn controls the substrate release rate to realize defined exponential growth during feed phase. By employing this system, fed-batch bioprocesses can be screened at high-throughput and with minimal manual effort. Therefore, our approach can significantly accelerate the scale-up of bioprocesses from lab scale to industrial scale.

Future work will focus on tackling the presented challenges of the current system. In particular, by employing the developed techniques, different feeding modes, e.g., constant feed rates or different slow-release systems can be realized. The feedback could then be used to account for enzyme instability or changing environmental conditions, which are the common drawbacks for enzyme-based slow-release systems. Furthermore, combining FeedER with already-established phenotyping technologies such as repeated low‐volume sampling [31] or proteomics [32] will lead to deep insights regarding the metabolism of potential producer strains at an early process stage. Another interesting extension would be the combination with automated adaptive laboratory evolution techniques [27], where untargeted strain development could be realized under carbon-limiting conditions.

## Electronic supplementary material

Below is the link to the electronic supplementary material.

Supplementary material 1 (DOCX 947 kb)
