# Supplementary material for: FeedER: a feedback-regulated enzyme-based slow-release system for fed-batch cultivation in microtiter plates
Source: Bioprocess Biosyst Eng. 2019 Aug 9;42(11):1843–52. doi: 10.1007/s00449-019-02180-z (PMC6800402; doi:10.1007/s00449-019-02180-z)
Supplement: Supplementary file 1 — Supplementary material 1 (DOCX 947 kb) [file 449_2019_2180_MOESM1_ESM.docx]

# Supplementary Material


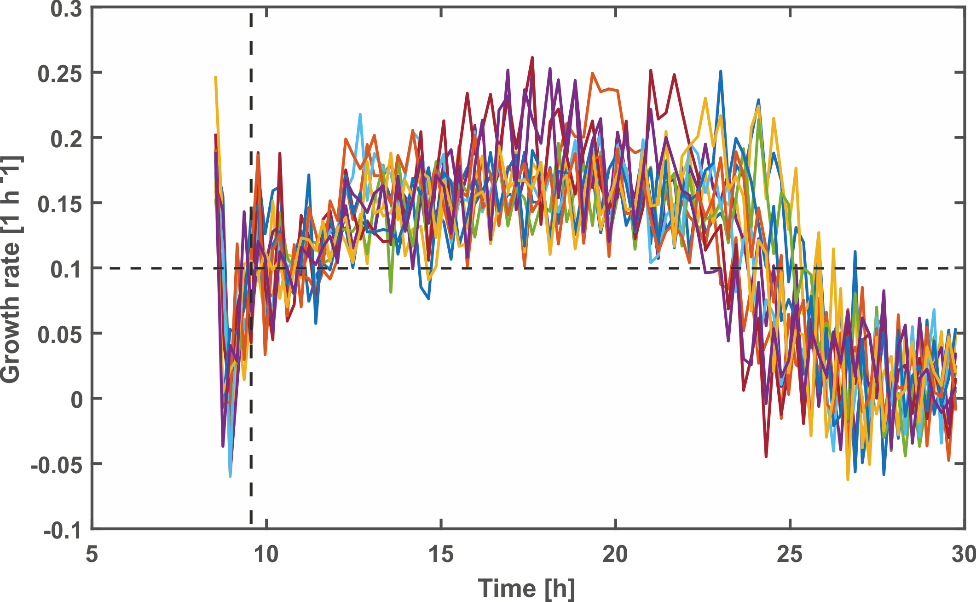


Fig. S1 Window calculation of the growth rate for 0.1 set point for all biological replicates. The well wise online calculated growth rate based on the online signals of all twelve cultivations. The addition of further enzyme, Amyloglucosidase was triggered, when the growth rate dropped below the predefined threshold of 0.1. Cultivations were performed in a 48-well FlowerPlate at 30°C, 1300 rpm, > 85 % humidity and the initial filling volume was 800 µL. Modified CGXII medium with 5 g L^-1^ d-glucose, a dextrin equivalent of 75 g L^-1^ d-glucose and 50 mM MOPS were used. The pH was kept constant at 7.1 through addition of 2.5 % (m v^-1^) NH_3_..


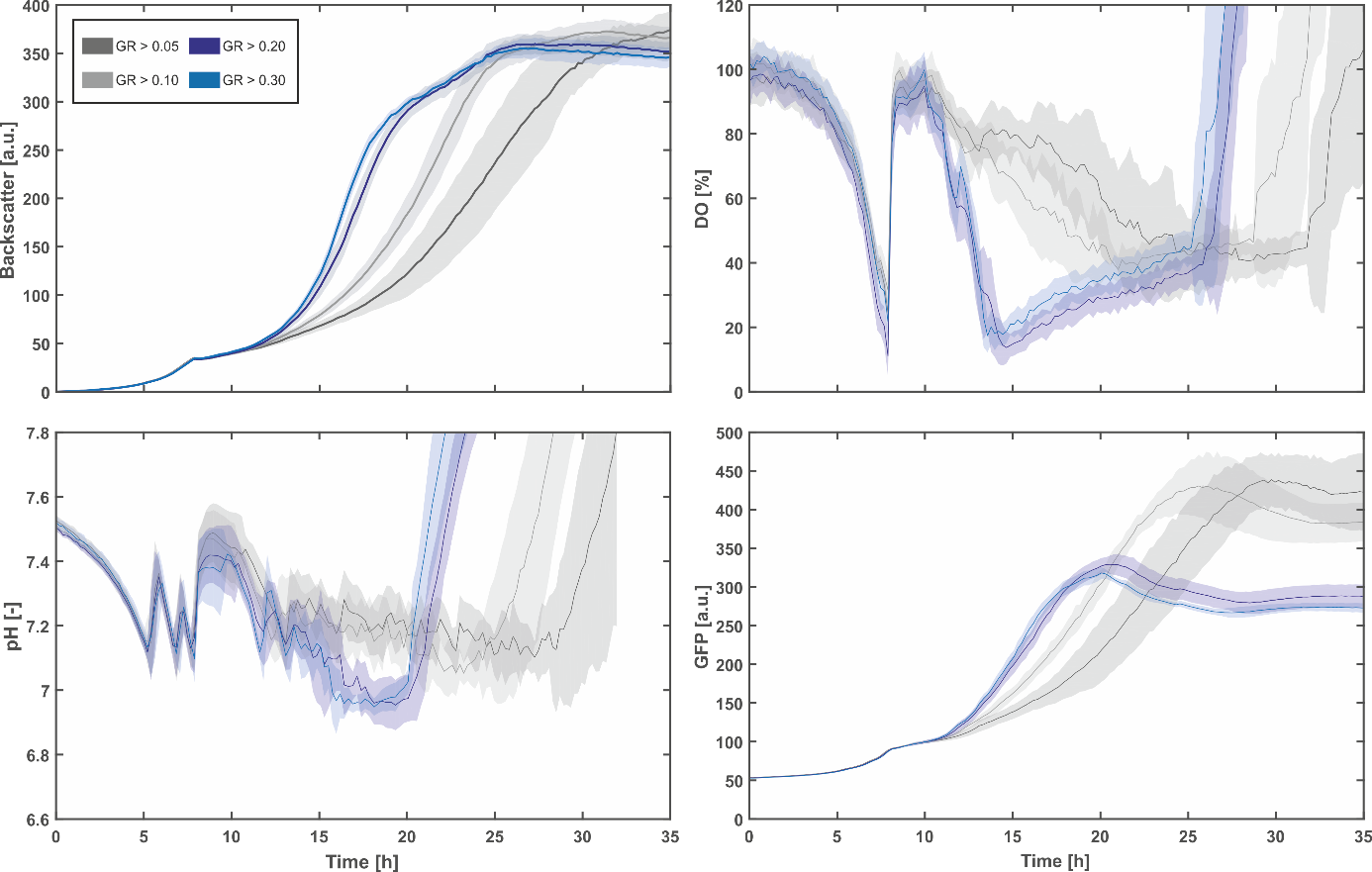


Fig. S2 Additional data for all fed batch cultivations. Cultivations were performed in a 48-well FlowerPlate at 30°C, 1300 rpm, > 85 % humidity and the initial filling volume was 800 µL. Modified CGXII medium with 5 g L^-1^ d-glucose, a dextrin equivalent of 75 g L^-1^ d-glucose and 50 mM MOPS were used. The pH was kept constant at 7.1 through addition of 2.5 % (m v^-1^) NH_3_. Four different growth rate set points *µ*_set_ = 0.05, 0.1, 0.2 and 0.3 h^-1^ were tested. The growth rates were kept above the set points through specific addition of Amyloglucosidase. Dark lines represent the arithmetic means of at twelve biological replicates and shaded areas represent corresponding standard deviations. The cultivations were stopped when a maximal filling volume of 1200 µL was reached.


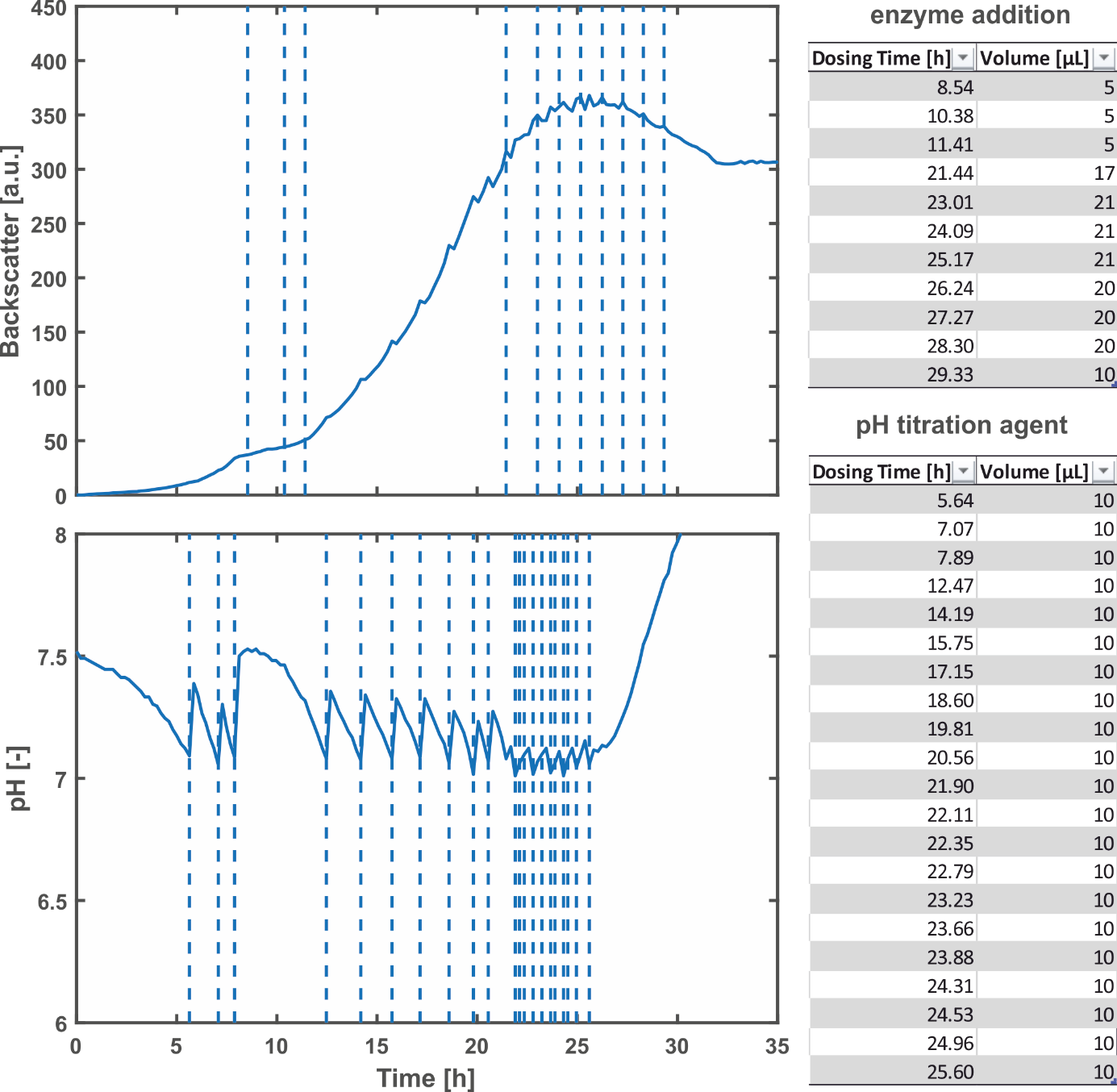


Fig. S3 Dosing history for an exemplary replicate from Figure 2. Each horizontal dashed line in the upper plot indicates the addition of enzyme, each horizontal dashed line in the lower plot indicates the addition of 2.5 % (m v^-1^) NH_3_ as titration agent. Information about the exact time points and volumes can be found in the tables next to the plots.


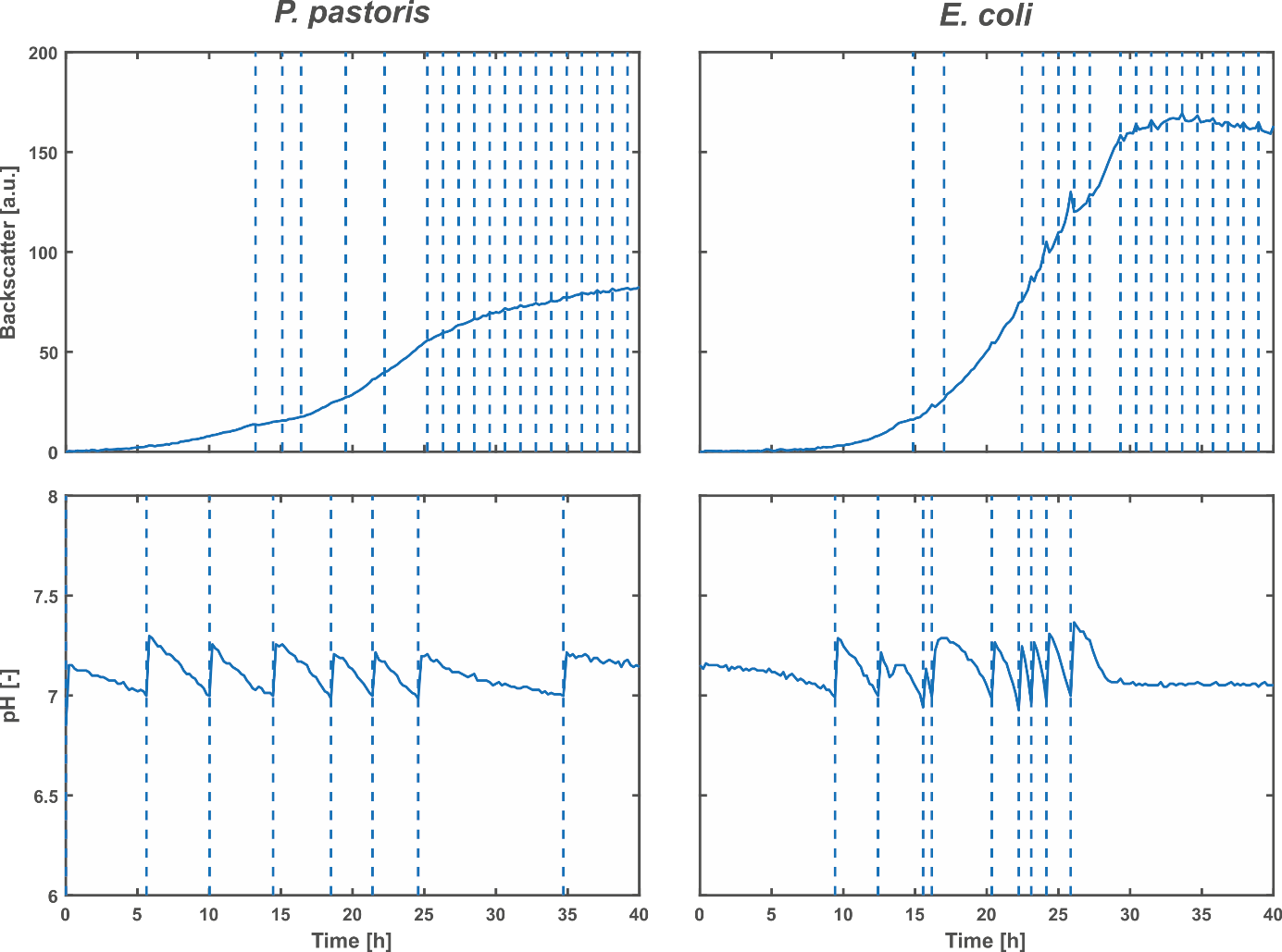


Fig. S4 Dosing history for exemplary replicates of Figure 3. Each horizontal dashed line in the upper plot indicates the addition of enzyme, each horizontal dashed line in the lower plot indicates the addition of 2.5 % (m v^-1^) NH_3_ as titration agent.
